# Endothelial Cells' Activation and Apoptosis Induced by a Subset of Antibodies against Human Cytomegalovirus: Relevance to the Pathogenesis of Atherosclerosis

**DOI:** 10.1371/journal.pone.0000473

**Published:** 2007-05-30

**Authors:** Claudio Lunardi, Marzia Dolcino, Dimitri Peterlana, Caterina Bason, Riccardo Navone, Nicola Tamassia, Elisa Tinazzi, Ruggero Beri, Roberto Corrocher, Antonio Puccetti

**Affiliations:** 1 Department of Clinical and Experimental Medicine, Section of Internal Medicine, University of Verona, Verona, Italy; 2 Institute Giannina Gaslini, Genova, Italy; 3 Department of Pathology, Section of General Pathology, University of Verona, Verona, Italy; 4 Department of Experimental Medicine, Section of Histology, University of Genova, Genova, Italy; Rockefeller University, United States of America

## Abstract

**Background:**

Human cytomegalovirus (hCMV) is involved in the pathogenesis of atherosclerosis. We have previously shown in patients with atherosclerosis that antibodies directed against the hCMV-derived proteins US28 and UL122 are able to induce endothelial cell damage and apoptosis of non-stressed endothelial cells through cross-rection with normally expressed surface molecules. Our aim was to dissect the molecular basis of such interaction and to investigate mechanisms linking innate immunity to atherosclerosis.

**Methodology/Principal Findings:**

We analysed the gene expression profiles in endothelial cells stimulated with antibodies affinity-purified against either the UL122 or the US28 peptides using the microarray technology. Microarray results were validated by quantitative PCR and by detection of proteins in the medium. Supernatant of endothelial cells incubated with antibodies was analysed also for the presence of Heat Shock Protein (HSP)60 and was used to assess stimulation of Toll-Like Receptor-4 (TLR4). Antibodies against UL122 and US28 induced the expression of genes encoding for adhesion molecules, chemokines, growth factors and molecules involved in the apoptotis process together with other genes known to be involved in the initiation and progression of the atherosclerotic process. HSP60 was released in the medium of cells incubated with anti-US28 antibodies and was able to engage TLR4.

**Conclusions/Significance:**

Antibodies directed against hCMV modulate the expression of genes coding for molecules involved in activation and apoptosis of endothelial cells, processes known to play a pivotal role in the pathogenesis of atherosclerosis. Moreover, endothelial cells exposed to such antibodies express HSP60 on the cell surface and release HSP60 in the medium able to activate TLR4. These data confirm that antibodies directed against hCMV-derived proteins US28 and UL122 purified from patients with coronary artery disease induce endothelial cell damage and support the hypothesis that hCMV infection may play a crucial role in mediating the atherosclerotic process.

## Introduction

Atherosclerosis is the primary cause of morbidity and mortality worldwide. It is a multifactorial disorder influenced by genetic [1] and environmental factors [2]. Indeed the genetic background is greatly influenced by classical environmental risk factors, such as cholesterol, diabetes, hypertension, obesity and smoking. In addition inflammation, infection and autoimmunity have been recently suggested to play a role in the pathogenesis of the disease [3]. Infectious agents seem to be involved in endothelium damage by inducing an autoimmune response to Heat Shock Proteins (HSPs) [4]. Antibodies against HSP60 are present in the majority of patients with coronary artery disease and their titre correlates with disease severity [5].

We have previously shown that autoantibodies against HSP60 are present in most atherosclerotic patients and that these autoantibodies are directed to an aminoacid sequence at position 153-163 of HSP60 that shows homology with two human cytomegalovirus (hCMV)-derived proteins, UL122 and US28 [ref. 6]. Anti-HCMV antibodies were present in a slightly higher percentage in CAD patients than in controls, however only the sera of patients with CAD showed antibodies against the identified viral peptides. Both viral peptides, UL122 and US28, show sequence similarity with molecules normally expressed on endothelial cell surface: US28 shows homology with integrin alpha 6 (CD49f) and UL122 with connexin 45 and CD151 [ref. 6]. IgG antibodies affinity purified against the HSP60 peptide from the sera of ten patients with coronary artery disease (CAD) bound endothelial cell derived HSP60, recombinant human HSP60 and recognized the hCMV-derived proteins UL122 and US28 [6]. Antibodies against the HSP60 peptide affinity purified from the serum samples of controls did not cross-react with the viral proteins. The IgG antibody component affinity purified against the viral peptides from the patients with CAD, recognized both the viral proteins and HSP60. The sera of patients with no evidence of CAD did not show antibodies against the viral peptides [6]. Antibodies directed against HSP60 and viral peptides bound endothelial cells upon interaction with the cell surface receptors sharing sequence homology with the viral peptides, through a mechanism of molecular mimicry, and induced apoptosis of non-stressed endothelial cells [6], considered a primary event in the pathogenesis of atherosclerosis [7], [8]. Moreover, HSP60 exposed on the surface of stressed endothelial cells could amplify the antibody aggression through cross-recognition of the anti-viral antibodies [6].

Taking advantage of the results previously obtained, we aimed at analyzing the molecular effects of the identified anti-hCMV antibodies on endothelial cells, using the DNA microarray approach, in order to ascertain whether the sets of genes modulated by anti-hCMV-derived proteins UL122 and US28 antibodies, are associated with the pathogenesis of atherosclerosis. We found indeed that most of the genes modulated are known to be associated with the atherosclerotic process. Moreover such antibodies induced release of HSP60 able to activate Toll-Like Receptor 4 (TLR4).

## Material and Methods

### Patients

The clinical characteristics of the ten patients with angiographic evidence of coronary artery disease, whose sera were used to obtain affinity purified antibodies against the peptides, have already been described elsewhere [6].

### Purification of anti-US28 and anti-UL122 IgG antibodies

The UL122-derived peptide (GPKKKSKRIS), the US28-derived peptide (TDVLNQSKPVT) and the irrelevant control peptide (VTLPKDSDVELP) chosen on the basis of its absence of relevant homology with the viral peptides and with the endothelial molecules connexin 45, CD49f and CD151, were synthesized by solid phase synthesis with the 9-fluorenylmethoxycarbonyl strategy [6].

To affinity purify IgG antibodies against either the UL122 or the US28 peptide or the control peptide, each peptide (5 mg/g of dried sepharose powder) was coupled to sepharose 4B (Pharmacia Uppsala, Sweden), according to the manufacturer's instructions. Pooled sera from the 10 different patients analysed were diluted in PBS and bound immunoglobulins were eluted with 0,1 M glycine pH 2,5 and dialysed against PBS [6].

### Cell culture and apoptosis

Human endothelial cells (HUVECs) and their growth media were purchased from Promocell Bioscience Alive (Heidelberg, Germany). HUVECs were used between passages 2 and 5. Cell monolayers were stimulated with antibodies purified against the UL122 or US28 peptide or the irrelevant control peptide (20 microgram/ml) and apoptosis evaluated at various intervals of time. Tumor necrosis factor alpha (TNFalpha 50 µg/L) was used as positive control to induce endothelial cell apoptosis. The extent of DNA fragmentation was quantified using a commercially available kit (Roche Biochemical, Basel, Switzerland). The enrichment of mono-and oligo-nucleosomes released into the cytoplasm is calculated as absorbance of sample cells (cells treated with antibodies)/absorbance of control cells (cells not exposed to antibodies). Enrichment factor is used as an index of apoptosis. An increase in the enrichment factor of 1.0 corresponded ap–proximately to 8–12% of apoptotic cells as determined in parallel by Fluorescence-activated cell sorting (FACS) analysis after staining with FITC-labeled annexin V (Alexis Biochemicals, San Diego, USA) (data not shown).

### Detection of HSP60 and TLR4 activation

For FACS analysis, we incubated cells with specific or control antibodies for 30 minutes on ice. Antibody binding was revealed using fluorescein isothiocyanate (FITC)-conjugated secondary antibodies. Samples were run on a FACScan flow cytometer (Becton Dickinson, Mountain View, CA).

Soluble HSP60 released in the supernatant was evaluated using a commercially available kit (Stressgen, Victoria, BC, Canada). Surface expression of HSP60 was assessed by FACS analysis using a monoclonal antibody directed against HSP60 (clone KL-1, Stressgen). HUVEC cells stressed with 0,1% glutaraldehyde for 20 minutes on ice were used as positive control.

TLR signaling leads to translocation of NF-kB. To monitor the induction of TLR4 signaling in response to ligand stimulation, we have used the pNifty reporter plasmid (Invivogen, Ruschlikon, Switzerland), expressing the secreted embryonic alkaline phosphatase gene (SEAP) under the control of a NF-kB-inducible ELAM1 composite promoter. Engineered 293T cells stably transfected with TLR4 and the co-receptors MD2 and CD14 (Invivogen) were co-transfected with the pNifty plasmids. Cells were grown to 60–80% confluence in growth medium and then harvested and resuspended in HEK-Blue Detection medium (Invivogen). This medium is specifically designed for detection of NF-kB activation since it turns blue in the presence of phosphatase activity. 2,5×10^4^ cells/well were plated in 96 well plates in the presence of the supernatant. Positive control for TLR4 activation was LPS (Invivogen). Supernatant was devoided of HSP60 using an anti-HSP60 monoclonal antibody (Stressgen) bound to a sepharose column. Cells were incubated for 24 hrs at 37°C in 5% CO_2_ and the blue color assessed by a spectrophotometer set at 620 nm. The supernatants used in this set of experiments were devoided of LPS contaminants using detoxy-gel endotoxin removing columns (Pierce Biotechnology, Rockford, IL, USA). Mock-transfected 293T cells were used as negative control.

### Gene Array analysis

Cell pellets of endothelial cells cultured as previously described and obtained after 6 and 12 hours of incubation with affinity purified antibodies against US28, UL122 and an irrelevant peptide, were used for gene array experiments. Preparation of cRNA, hybridization, and scanning of probe arrays were performed according to the protocols of the manufacturer (Affymetrix Santa Clara, California, United States) by the Genopolis Consortium (University of Milano-Bicocca, Italy) using the Human Genome U133A GeneChip^®^ (Affymetrix). The GeneChip^®^ Human Genome U133A is a single array representing 14,500 well-characterized human genes and including more than 22,000 probe sets and 500,000 distinct oligonucleotide features.

The different gene expression patterns were analyzed using the Array Assist software version 2.0 (Stratagene, La Jolla, California, United States) that calculated a robust multi-array average of background-adjusted, normalized, and log-transformed intensity values applying the Robust Multi-Array Average algorithm (RMA). With this software the mean optical background level for each array was subtracted from the signal intensity for each probe. Finally, the normalized, background-corrected data were transformed to the log_2_ scale. A Signal Log_2_ Ratio of 1.0 indicates an increase of the transcript level by two fold change (2 F.C.) and−1.0 indicates a decrease by two fold (−2 F.C.). A Signal Log Ratio of zero would indicate no change [9].

In our study, we analyzed the gene expression profiles in endothelial cells stimulated with antibodies purified against either the UL122 or the US28 peptide (test samples) or with antibodies purified against an irrelevant peptide (control samples) for 6 and 12 hours. Genes were selected for final consideration when their expression (F.C.) was at least 1.5-fold different in the test sample versus control sample at least at one time point. Experiments were performed in duplicates. The microarray results have been reported according to the MIAME guidelines and deposited in the public repository Array-Express at http://www.ebi.ac.uk/arrayexpress.

To verify relationships from our gene list and the existing knowledge in the literature concerning genes and atherosclerosis, we used the automated literature mining tool, termed MedGene (hptt://hipseq.med.harvard.edu/MEDGENE/login.jsp), which summarizes and estimates the relative strength of all human gene disease relationships in Medline [10].

The software package Pathway Studio (Ariadne Genomics, Rockville USA) was used to identify functional interrelationships among the genes identified. These functional relationships were then graphically represented by the software as a network.

### Validation of Gene Array results

Quantitative real-time polymerase chain reaction (Q-PCR) was carried out as described [9]. Briefly, total RNA has been extracted from HUVECs with TRIzol reagent (Invitrogen, Carlsbad, CA, USA), following manufacturer's instructions. One µg of total RNA from each sample was treated with amplification grade DNase I and then used as a template for the reverse transcription reaction, using random hexamers and SuperScript II Reverse Transcriptase (Invitrogen). All samples will be reverse transcribed under the same conditions and from the same reverse transcription master mix, in order to minimize differences in reverse transcription efficiency. Triplicate Q-PCR reactions for each sample have been performed. Control wells containing no template have been used to exclude the presence of contaminating template molecules and to identify potential primer-dimer products from the dissociation curve analysis. Amplification plots have been analyzed using Opticon Monitor software version 2.02 (MJ Research, Waltham, MA, USA) and data calculated with Q-Gene software (www.BioTechniques.com) and expressed as mean normalized expression (MNE). Glyceraldehyde-3-phosphate dehydrogenase (GAPDH) has been used as a normalizing gene according to its stable expression levels.

Detection of MCP-1 (Amersham Biosciences, Pisacataway, NJ, USA) and sE-Selectin (R&D Systems, Minneapolis, Minnesota, USA) in the supernatants were determined by ELISA.

### Statistical analysis

Statistical testing was performed using StatsDirect (StatDirect, Cheshire, UK). Difference in the concentration of soluble molecules in the supernatants was performed using a parametric test. *P*-values less than 0.05 was considered significant.

## Results

**Figure 1:**
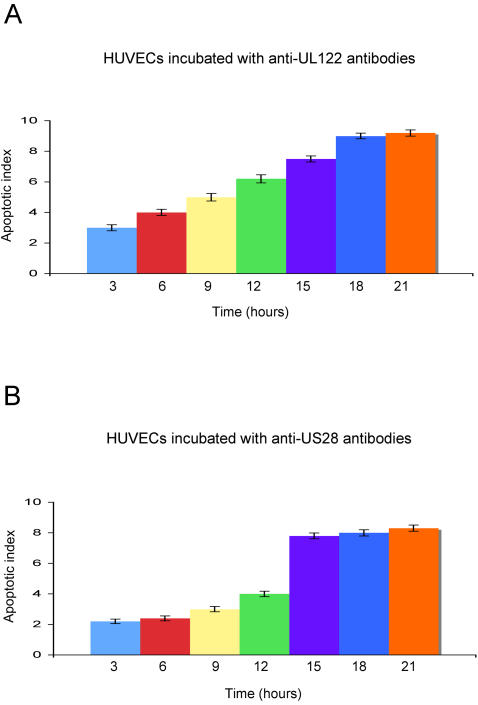
Anti-hCMV antibodies induce apoptosis of endothelial cells. Apoptosis induced in HUVECs by antibodies directed against UL122 peptide (A) and against US28 peptide (B); X axis: apoptotic index; Y axis: time expressed in hours. Data are mean of three independent experiments. Error bars = SD. Apoptosis induced by antibodies directed against the irrelevant peptide gave an apoptotic index<1 at all time points, corresponding to less than 8–12% of apoptotic cells as determined in parallel by FACS analysis.

**Figure 2:**
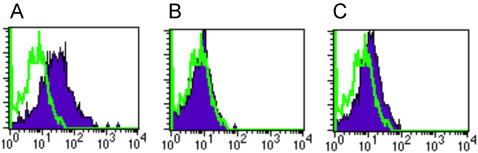
FACS analysis of the expression of HSP60 at the cell surface membrane. (A) Positive control: HUVECs stressed with glutaraldehyde for 20 minutes; (B) HUVECs treated with anti-UL122 antibodies for 9 hours; (C) HUVECs treated with anti-US 28 antibodies for 9 hours. HUVEC cells were stained with a monoclonal antibody directed against HSP60. Percentage of positive cells: (A) 44,2%; (B) 1,3%; (C): 6,7%.

**Figure 3:**
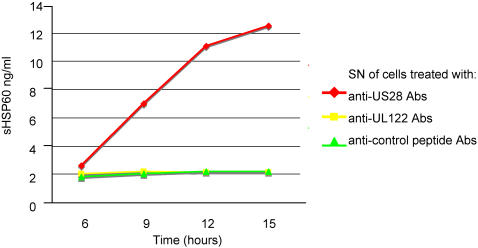
Soluble HSP60 in the supernatant of stimulated cells. Soluble HSP60 in the supernatant (SN) of HUVECs after stimulation with anti-US28 and anti-UL122 antibodies (Abs) at different time points. X axis: time expressed in hours. Y axis: soluble HSP60 concentration expressed in ng/ml.

The IgG antibodies affinity purified against the HSP60 and the viral peptides obtained from patients with CAD had the same behaviour of the purified antibodies previously described both in the recognition of endothelial cell surface molecules and in the ability to induce endothelial cell apoptosis [6]. To more precisely analyze the mechanisms by which anti-hCMV-derived proteins UL122 and US28 antibodies induce apoptosis of HUVECs [6], a time course experiment was performed by incubating HUVECs with either anti-UL122 or anti-US28 or anti-irrelevant peptide antibodies obtained from the 10 patients with CAD. Figure 1, panels A and B, shows that apoptosis induced by anti-UL122 antibodies occurs at an earlier stage compared to the other anti-hCMV antibody subset. Moreover internucleosomal DNA fragmentation induced by anti-UL122 peptide antibodies reached 80% and that induced by anti-US28 reached 72% of the level of internucleosomal DNA fragmentation seen after exposure of endothelial cells to 50 µg/L of TNFalpha. This finding is in accordance with our previous report that the antibodies can trigger apoptosis in human endothelial cells upon interaction with different cell-membrane molecules through a mechanism of molecular mimicry [6]. Antibodies against HSP60 play a pivotal role in the cytotoxic damage of stressed endothelial cells; since antibodies against hCMV are able to crossreact with particular HSP60 epitopes thus amplifying the cell damage by binding surface HSP60, we evaluated the surface HSP60 expression in endothelial cells after exposure to anti-UL122 and US28 antibodies. Surprisingly only HUVECs incubated with anti-US28 antibodies translocated HSP60 on the cell surface (figure 2, panels A–C) and released the soluble molecule (sHSP60) in the medium (figure 3). These data suggest that the two antibody subsets induce apoptosis through different mechanisms: anti-UL122 antibodies induce apoptosis through the engagement of the surface molecules CD151 and connexin 45 (GJA7), whereas the apoptotic damage triggered by the engagement of CD49f (ITGA6) by anti-US28 can be further amplified by the antibody interaction with HSP60 expressed on the cell surface.

**Figure 4:**
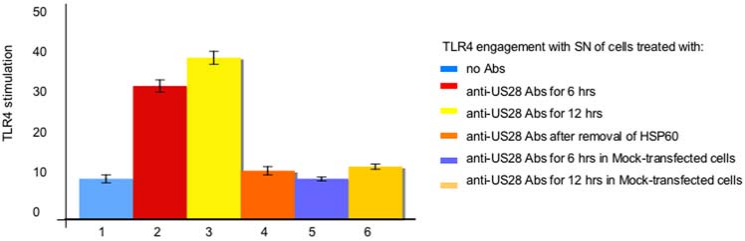
TLR4 activation by the supernatant of treated cells. TLR4 engagement by the SN of cells treated with the antibodies (Abs) described. Y axis: levels of TLR4 stimulation expressed as ng/mL of LPS. Data are mean of three independent experiments. Error bars = SD.

Since a link between innate immunity and atherosclerosis has been proposed and several data indicate that signaling through TLR4 promotes atherosclerosis [11], and since HSP60 has been proposed as an endogenous ligand for TLR4 [12], we next evaluated whether HSP60 present in the supernatant of endothelial cells stimulated with anti-US28 antibodies could engage TLR4. 293T cells transfected with TLR4 were activated by the supernatant that induced an activation of TLR4 similar to the one obtained with 40 ng of LPS (figure 4). Such activation was not present when the supernatant was deprived of HSP60 and when supernatant was used with mock-transfected 293T cells. These data suggest that HSP60 is indeed an endogenously generated ligand for TLR4.

**Figure 5:**
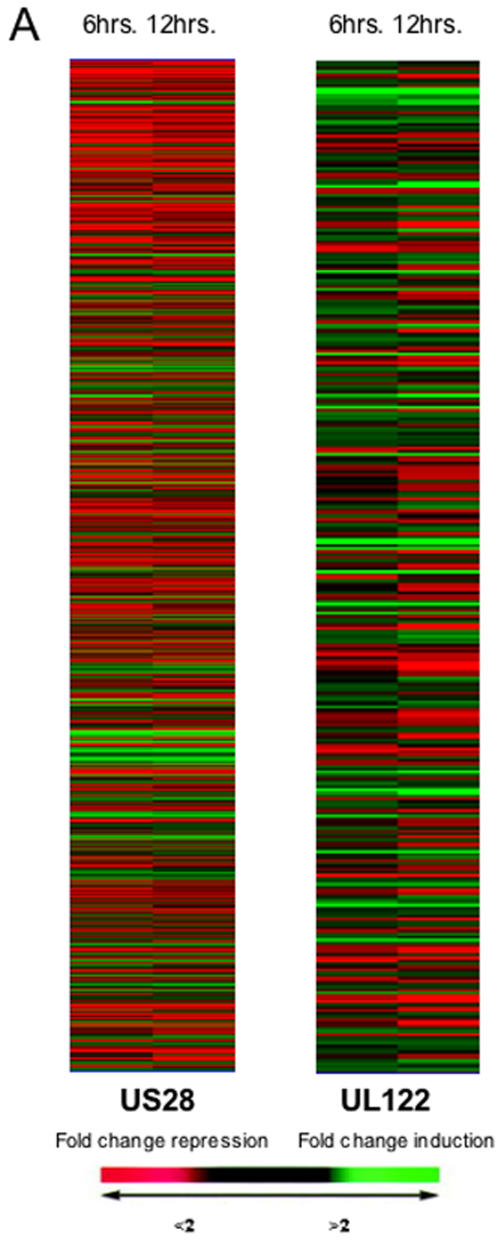
Gene array analysis of HUVECs stimulated with anti-hCMV antibodies. Clustergrams of genes modulated by incubation of endothelial cells with antibodies against the US28 peptide or the UL122 peptide. Colours indicate the fold change.

We next analyzed the gene expression profiles in endothelial cells treated with antibodies affinity purified against the hCMV-derived peptides in order to identify clusters of genes involved in the pathogenesis of vascular damage in atherosclerosis. Anti-US28 peptide antibodies up-regulated 907 transcripts (Table 1 and Table S1). Clustergrams of modulated genes are shown in figure 5. Anti-US28 antibodies up-regulated genes encoding for adhesion molecules, such as E selectin, VCAM-1 and ICAM-1 and genes encoding for chemokines including CCL2, CCL20, CXCL1, CXCL2, together with chemokine receptors such as CCR3, CXCR4.

**Table 1:** Gene expression in HUVEC after 6 and 12 huors of stimulation with anti-US28 IgG antibodies

Gene accession	Gene description	Gene Symbol	F.C. 6 h	F.C.12 h
**Adhesion molecules**				
NM_000450.1	endothelial adhesion molecule 1	SELE	5,95	1,79
NM_001078.2	vascular cell adhesion molecule 1	VCAM1	3,76	2,06
NM_002203.2	integrin, alpha 2	ITGA2	2,61	2,69
NM_000212.2	integrin, beta 3	ITGB3	1,72	1,72
NM_000201.1	intercellular adhesion molecule 1 (CD54)	ICAM1	1,60	1,33
**Inflammation**				
S69738.1	chemokine (C-C motif) ligand 2	CCL2	2,46	1,98
NM_004591.1	chemokine (C-C motif) ligand 20	CCL20	1,63	1,42
NM_001511.1	chemokine (C-X-C motif) ligand 1	CXCL1	3,32	1,63
M57731.1	chemokine (C-X-C motif) ligand 2	CXCL2	4,21	2,72
NM_002090.2	chemokine (C-X-C motif) ligand 3	CXCL3	1,62	1,40
NM_001837.2	chemokine (C-C motif) receptor 3	CCR3	1,49	1,77
AJ224869	chemokine (C-X-C motif) receptor 4	CXCR4	2,24	1,99
NM_017801.2	chemokine-like factor super family 6	CKLFSF6	2,07	1,69
NM_003326.2	tumor necrosis factor (ligand) superfamily, member 4	TNFSF4	1,96	1,36
NM_002184.2	interleukin 6 signal transducer	IL6ST	3,61	4,36
NM_002185.2	interleukin 7 receptor	IL7R	2,04	2,01
**Immune responce**				
NM_000593.3	transporter 1, ATP-binding cassette, sub-family B	TAP1	1,98	1,85
NM_005348.2	heat shock 90kDa protein 1, alpha	HSPCA	2,11	1,84
NM_006260.2	DnaJ (Hsp40) homolog, subfamily C, member 3	DNAJC3	1,78	1,65
**Transcription**				
NM_005239.4	v-ets erythroblastosis virus E26 oncogene homolog 2	ETS2	3,21	2,34
NM_003199.1	transcription factor 4	TCF4	3,12	3,42
NM_021964.1	zinc finger protein 148	ZNF148	3,53	2,92
BC003566.1	zinc finger protein 24	ZNF24	3,44	2,76
BC003070.2	GATA binding protein 3	GATA3	1,91	1,50
NM_007348.2	activating transcription factor 6	ATF6	2,79	2,69
U97103.1	alpha thalassemia/mental retardation syndrome X-linked	ATRX	4,68	4,41
NM_013448.2	bromodomain adjacent to zinc finger domain, 1A	BAZ1A	3,98	4,62
NM_013354.5	CCR4-NOT transcription complex, subunit 7	CNOT7	1,71	1,60
NM_004459.5	fetal Alzheimer antigen	FALZ	2,97	3,00
AF548661	general transcription factor IIH, polypeptide 3, 34kDa	GTF2H3	3,33	3,68
NM_003489.2	nuclear receptor interacting protein 1	NRIP1	11,86	8,12
NM_004774.2	PPAR binding protein	PPARBP	2,88	2,67
**Inhibition of cell growth and apoptosis**				
NM_001970.3	eukaryotic translation initiation factor 5A	EIF5A	22,93	17,78
BC000076.2	cyclin D1 (PRAD1: parathyroid adenomatosis 1)	CCND1	2,03	2,04
NM_003810.2	tumor necrosis factor (ligand) superfamily, member 10	TNFSF10	2,03	2,53
U20537.1	caspase 6, apoptosis-related cysteine protease	CASP6	2,65	2,35
NM_001227.3	caspase 7, apoptosis-related cysteine protease	CASP7	1,61	1,31
NM_005256.2	growth arrest-specific 2	GAS2	1,84	1,19
BC001152.1	growth arrest-specific 7	GAS7	1,78	1,95
NM_004401.2	DNA fragmentation factor, 45kDa, alpha polypeptide	DFFA	1,23	1,66
NM_020529.1	nuclear factor of kappa light polypeptide gene enhancer in B-cells inhibitor, alpha	NFKBIA	2,40	1,75
AF091555.1	C-terminal binding protein 1	CTBP1	6,48	5,10
NM_021111.1	reversion-inducing-cysteine-rich protein with kazal motifs	RECK	1,74	1,74
NM_016272.3	transducer of ERBB2, 2	TOB2	1,92	1,99
BC006325.2	G-2 and S-phase expressed 1	GTSE1	1,92	1,92
**Collagens and extracellular matrix**				
NM_005713.1	collagen, type IV, alpha 3 binding protein	COL4A3BP	1,76	1,87
NM_006665.2	heparanase	HPSE	1,74	1,33
NM_021809.4	TGFB-induced factor 2 (TALE family homeobox)	TGIF2	1,69	1,13
NM_003243.2	transforming growth factor, beta receptor III (betaglycan)	TGFBR3	1,55	1,61
**Metabolism**				
NM_014508.2	apolipoprotein B mRNA editing enzyme, catalytic polypeptide-like 3C	APOBEC3C	1,88	2,00
BC015818.1	lectin, galactoside-binding, soluble, 8 (galectin 8)	LGALS8	2,78	1,38
NM_002858.2	ATP-binding cassette, sub-family D (ALD), member 3	ABCD3	1,86	1,50
NM_017526.2	leptin receptor	LEPR	1,74	1,98
AF285167.1	ATP-binding cassette, sub-family A,1	ABCA1	1,52	1,11
NM_005506.2	scavenger receptor class B, member 2	SCARB2	1,42	1,88
**Growth factors**				
NM_002632.4	placental growth factor	PGF	3,34	3,02
**Receptors**				
NM_000875.2	insulin-like growth factor 1 receptor	IGF1R	4,41	3,99
AW157070	epidermal growth factor receptor precursor	EGFR	3,11	2,57
M34641	fibroblast growth factor receptor 1	FGFR1	2,47	2,61
NM_000565.2	interleukin 6 receptor	IL6R	1,55	1,31
NM_000808.2	gamma-aminobutyric acid (GABA) A receptor, alpha 3	GABRA3	1,43	1,58
**Enzymes**				
NM_003358.1	UDP-glucose ceramide glucosyltransferase	UGCG	3,50	2,73
AF065214.1	phospholipase A2, group IVC (cytosolic, calcium-independent)	PLA2G4C	3,36	3,64
NM_001394.5	dual specificity phosphatase 4	DUSP4	3,06	2,69
NM_024906.1	stearoyl-CoA desaturase 4	SCD4	2,45	1,57
NM_006888.2	calmodulin 1	CALM1	5,48	5,56
M61906.1	phosphoinositide-3-kinase, regulatory subunit (p85 alpha)	PIK3R1	2,25	2,31
L18964.1	protein kinase C, iota	PRKCI	2,21	2,60
BC005365.1	mitogen-activated protein kinase kinase 7	MAP2K7	2	2,45
**Solute carriers**				
NM_000112.2	solute carrier family 26 (sulfate transporter), member 2	SLC26A2	5,70	4,53
NM_004731.3	solute carrier family 16 (monocarboxylic acid transporters), member 7	SLC16A7	2,92	2,11
**Small G proteins**				
AI189609	RAB2, member RAS oncogene family	RAB2	2,64	2,93
NM_022157.2	Ras-related GTP binding C	RRAGC	2,19	1,25
**Miscellaneuos**				
NM_001015.3	ribosomal protein S11	RPS11	119,98	40,70
BC000603.2	ribosomal protein L38	RPL38	31,39	15,66
BC000023.2	ribosomal protein S19	RPS19	3,08	3,08
M25077	Sjogren syndrome antigen A2 (60kDa, ribonucleoprotein autoantigen SS-A/Ro)	SSA2	2,38	2,12
AF055585.1	slit 2	SLIT2	2,16	1,66
NM_014399.3	transmembrane 4 superfamily member 13	TM4SF13	1,56	1,60
AF065241.1	thioredoxin (stress-induced)	TXN	2,43	2,04
NM_006287.3	tissue factor inhibitor protein	TFPI	6,64	7,77
NM_033360.2	v-Ki-ras2 Kirsten rat sarcoma 2 viral oncogene homolog	KRAS2	8,92	8,48

Genes encoding for molecules involved in the apoptotic process were induced including eukaryotic translation initiation factor 5 (EIF5A) [13], which induces the expression of proteins essential for apoptosis of HUVECs, and other apoptosis-related genes such as caspase-6 (CASP6) and CASP7 and DNA fragmentation factor (DFFA). Up-regulated transcription factors included transcription factor 4 (TCF4) and v-ets erythroblastosis virus E26 oncogene homolog 2 (ETS2), involved in promoting apoptosis [14]. Moreover some ribosomal proteins were highly induced (up to 119,98 fold change in expression for RPS11) as already observed in HUVECs treated with asymmetrical dimethylarginine (ADMA), an endogenous inhibitor of nitric oxide synthase considered a risk factor for cardiovascular disease [15]. Interestingly ribosomal protein S19 is over-expressed during apoptotic cell death [16]. Genes involved in the mitogen-activated protein kinase (MAPK) signal pathway, which plays a pitoval role in transmitting transmembrane signals required for cell proliferation and apoptosis, were up-regulated by anti-US28 antibodies, including MAP2K7 (or MKK7), one of the major SAPK/JNK activating kinases [17], calmodulin 1 (CALM1) [18] and phosphatidylinositol 3-kinase regulatory 1 (PIK3R1). The activation of the MAPK pathway is responsible of the activation of other regulatory proteins, including the cytosolic enzyme phospholipase A2 (PLA2G4C), upregulated by anti-US28 antibodies, known to be induced in HUVECs in response to hypoxia, with consequent release of arachidonic acid [19].

Induction of growth factors is a common feature of endothelial cell activation; interestingly placental growth factor (PGF), known to promote atherosclerotic intimal thickening and macrophage accumulation [20], was induced by anti-US28 peptide antibodies. Moreover epidermal growth factor receptor (EGFR) and fibroblast growth factor receptor 1 (FGFR1), both induced by anti-US28 antibodies, are expressed in atherosclerotic plaques [21], [22]. Finally, several other genes were up-regulated including genes involved in lipid metabolism such as leptin receptor (LEPR), ATP-binding cassette (ABCA1) and peroxisome proliferator-activated receptor binding protein (PPARBP), and in extracellular matrix metabolism: collagen type IV alpha 3 binding protein (COL4A3BP), heparanase (HPSE), TGF beta-induced factor 2 (TGIF2).

Among the 1379 transcripts down-regulated by anti-US28 antibodies (Table S2) there were endothelial nitric oxide synthase (eNOS) and LDL receptor. The gene encoding for the anti-apoptotic molecule programmed cell death 4 protein (PDCD4) was repressed, accordingly to the observation that endothelial cells undergo apoptosis. Interestingly we could observe a down-regulation of HSP70, reported to exert a protective role against the development of atherosclerosis [23].

Anti-UL122 peptide antibodies up-regulated 186 transcripts including genes encoding for adhesion molecules, chemokines, transcription factors and molecules involved in apoptosis (Table 2 and Table S3). The highest increase in expression was observed among genes encoding for adhesion molecules: selectin E, VCAM-1 and ICAM-1. Another cluster of up-regulated genes was represented by genes encoding for inflammatory molecules including chemokines; CCL2, was highly increased, with a peak of 19-fold increase in expression at 6 hrs of stimulation. Others chemokine genes were highly induced, including CCL20, CXCL2, CXCL3 and CX3CL1. Genes encoding for extracellular matrix and matrix remodeling proteins [24] were also found up-regulated: matrix metalloproteinase-10 (MMP-10), laminin, nidogen, collagen type IV and cathepsin S. Several genes encoding for molecules involved in apoptosis such as CASP7, tumor necrosis factor alpha-induced protein 1 (TNFAIP1), and TNFAIP2 were upregulated. Similarly to what observed with anti-US28 peptide antibodies, anti-UL122 antibodies induced up-regulation of transcription factors, in particular genes involved in NF-kB activation pathway such as TANK and RELB, which have been implicated in atherosclerosis [25]. Moreover genes encoding for a variety of proteins that have been associated with atherosclerosis were up-regulated including phospholipase A2 (PLA2G4C), prostaglandin synthase 2 (PTGS2) also known as cyclooxygenase-2, thrombin receptor like-1 and the gene encoding for superoxide dismutase 2 (SOD2).

**Table 2:** Gene expression in HUVEC after 6 and 12 hours of stimulation with anti-UL122 IgG antibodies

Gene accession	Gene description	Gene Symbol	F.C. 6 h	F.C.12 h
**Adhesion molecules**				
NM_000450.1	selectin E (endothelial adhesion molecule 1)	SELE	31,15	15
NM_001078.2	vascular cell adhesion molecule 1	VCAM1	18,8	6,1
NM_000201.1	intercellular adhesion molecule 1 (CD54)	ICAM-1	12,7	7,2
NM_002210.2	integrin, alpha V	ITGAV	1,54	1,32
**Inflammation**				
S69738.1	chemokine (C-C motif) ligand 2	CCL2	19	16
NM_004591.1	chemokine (C-C motif) ligand 20	CCL20	7,6	4,7
NM_001511.1	chemokine (C-X-C motif) ligand 1	CXCL1	6,2	4,5
M57731.1	chemokine (C-X-C motif) ligand 2	CXCL2	9,5	6,8
NM_002090.2	chemokine (C-X-C motif) ligand 3	CXCL3	10	6
NM_002994.3	chemokine (C-X-C motif) ligand 5	CXCL5	2	2
NM_002993.2	chemokine (C-X-C motif) ligand 6	CXCL6	2	1,8
NM_000584.2	interleukin 8	IL8	2,8	2,3
NM_005409.3	chemokine (C-X-C motif) ligand 11	CXCL11	5,5	6
NM_002996.3	chemokine (C-X3-C motif) ligand 1	CX3CL1	3,11	2,9
NM_002341.1	lymphotoxin beta (TNF superfamily, member 3)	LTB	1,67	1,6
NM_005658.3	TNF receptor-associated factor 1 (TRAF1)	TRAF1	1,6	1,37
NM_003300.2	TNF receptor-associated factor 3 (TRAF3)	TRAF 3	1,6	1,45
NM_000395.1	colony stimulating factor 2 receptor, beta	CSF2RB	1,52	1,2
AJ224869	chemokine (C-X-C motif) receptor 4	CXCR4	1,83	2,17
NM_004221.4	natural killer cell transcript 4 (interleukin 32)	NK4	1,6	4,3
NM_003855.2	interleukin 18 receptor 1	IL18R	2,02	1,3
**Immune response**				
X56841.1	major histocompatibility complex, class I, E	HLA-E	1,2	1,5
NM_000593.5	transporter 1, ATP-binding cassette, sub-family B	TAP 1	1,8	2,14
NM_006260.2	DnaJ (Hsp40) homolog, subfamily C, member 3	DNAJC3	1,19	1,63
**Transcription**				
NM_003440.2	zinc finger protein 140	ZNF140	1,6	1,3
NM_002448.2	msh homeo box homolog 1	MSX1	1,8	1,7
NM_006509.2	v-rel reticuloendotheliosis viral oncogene homolog B	RELB	2	2
NM_003199.1	transcription factor 4	TCF4	1,6	1,5
NM_030751.2	transcription factor 8	TCF 8	1,4	1,8
NM_004556.2	nuclear factor of kappa light polypeptide gene enhancer in B-cells inhibitor, epsilon	NFKBIE	2	1,8
NM_000222.1	v-kit Hardy-Zuckerman 4 feline sarcoma viral oncogene	KIT	2,3	2,2
U59863.1	TRAF family member associated NFKB activator	TANK	1,6	1,4
NM_005239.4	v-ets erythroblastosis virus E26 oncogene homolog 2	ETS2	1,26	1,53
NM_003489.2	nuclear receptor interacting protein 1	NRIP1	1,67	1,60
**Inhibition of cell growth and apoptosis**				
NM_006291.2	tumor necrosis factor, alpha-induced protein 2	TNFAIP2	2,37	1,57
NM_021137.3	tumor necrosis factor, alpha-induced protein 1 (endothelial)	TNFAIP1	1,7	1,44
NM_001227.3	caspase 7, apoptosis-related cysteine protease	CAS P7	1,68	1,35
NM_000546.2	tumor protein p53	TP53	1,2	1,7
NM_006290.2	tumor necrosis factor, alpha-induced protein 3	TNFAIP3	2,7	2,44
NM_003810.2	tumor necrosis factor (ligand) superfamily, member 10	TNFSF10	2,3	3,39
NM_020529.1	nuclear factor of kappa light polypeptide gene enhancer in B-cells inhibitor	NKFBIA	2,7	2,3
**Collagens and extra-cellular matrix**				
NM_007361.2	nidogen 2 (osteonidogen)	NID2	1,8	2,9
NM_001846.1	collagen, type IV, alpha 2	COL4A2	1,5	2,4
NM_001845.3	collagen, type IV, alpha 1	COL4A1	1,75	1,85
NM_002999.2	syndecan 4	SDC4	1,5	1,36
NM_004079.3	cathepsin S	CTSS	1,2	1,58
NM_002425.1	matrix metalloproteinase 10	MMP10	1,7	2,3
NM_005562.1	laminin,gamma 2	LAMC2	1,5	2,3
**Metabolism**				
NM_014349.2	apolipoprotein L, 3	APOL3	2,4	1,7
AF065214.1	phospholipase A2	PLA2 G4C	5,1	10,8
NM_004915.3	ATP-binding cassette, sub-family G (WHITE), member 1	ABCG1	3	2,8
**Miscellaneous**				
L07555.1	CD69 antigen	CD 69	6	3,7
NM_004428.2	ephrin-A1	EFNA1	2,1	1,8
NM_000963.1	prostaglandin-endoperoxide synthase 2 (COX-2)	PTGS2	1,9	1,3
NM_005242.3	Thrombin receptor like 1	F2RL1	1,72	1,63
NM_003277.2	Claudin 5	CLDN5	1,17	1,7
AY267901.1	superoxide dismutase 2, mitochondrial	SOD 2	5,5	4,6
NM_021804.1	angiotensin I converting enzyme (peptidyl-dipeptidase A) 2	ACE2	1,2	1,5
NM_006398.2	ubiquitin D	UBD	2,7	3,8
NM_002053.1	guanylate binding protein 1, interferon-inducible, 67kDa	GBP1	1,8	1,7

A total number of 140 transcripts were down-regulated by anti-UL122 antibodies (Table S4). Some genes are involved in lipid metabolism: 24-dehydrocholesterol reductase (DHCR24), squalene epoxidase (SQLE), LDL receptor and low density lipoprotein receptor-related protein 8 (LRP8). The decreased expression of the gene encoding for eNOS has already been reported in endothelial cells isolated from patients with vascular damage indicating an intrinsic defect in the mechanism of nitric oxide production [26].

**Figure 6:**
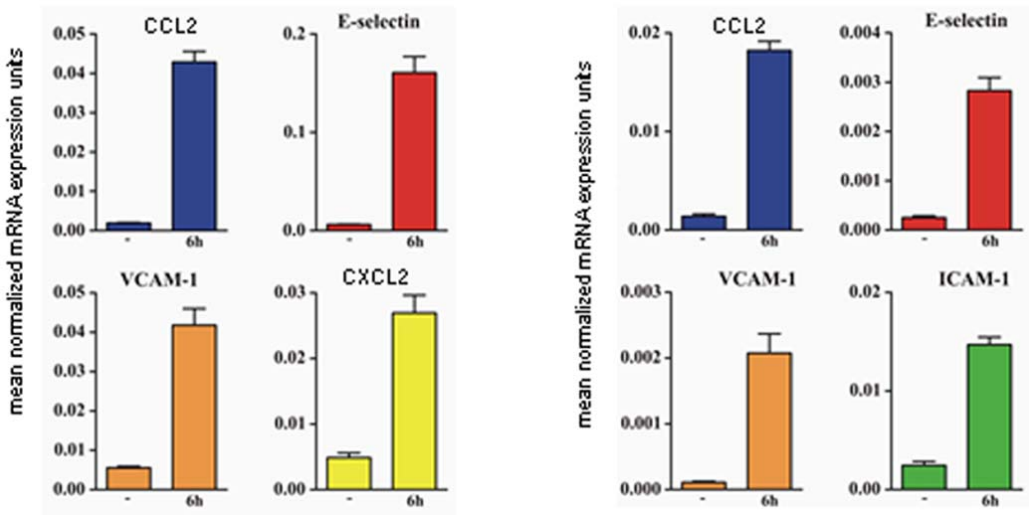
Validation of gene array by Q-PCR. (A) Genes selected for validation by Q-PCR in endothelial cells treated with anti-US28 peptide antibodies. CCL2, E-selectin, VCAM-1 and CXCL2 transcripts were increased by 2,46-, 5,95-, 3,76-, and 4,21-fold, respectively, compared with control endothelial cells. (B) Genes selected for validation by Q-PCR in endothelial cells treated with anti-UL122 antibodies. CCL2, E-selectin, VCAM-1 and ICAM-1 transcripts were increased 19-, 31,15-, 18,8-, and 12,7-fold compared to control endothelial cells. The level of transcript expression is reported on the vertical axis. *GAPDH* was selected as endogenous gene.

**Figure 7:**
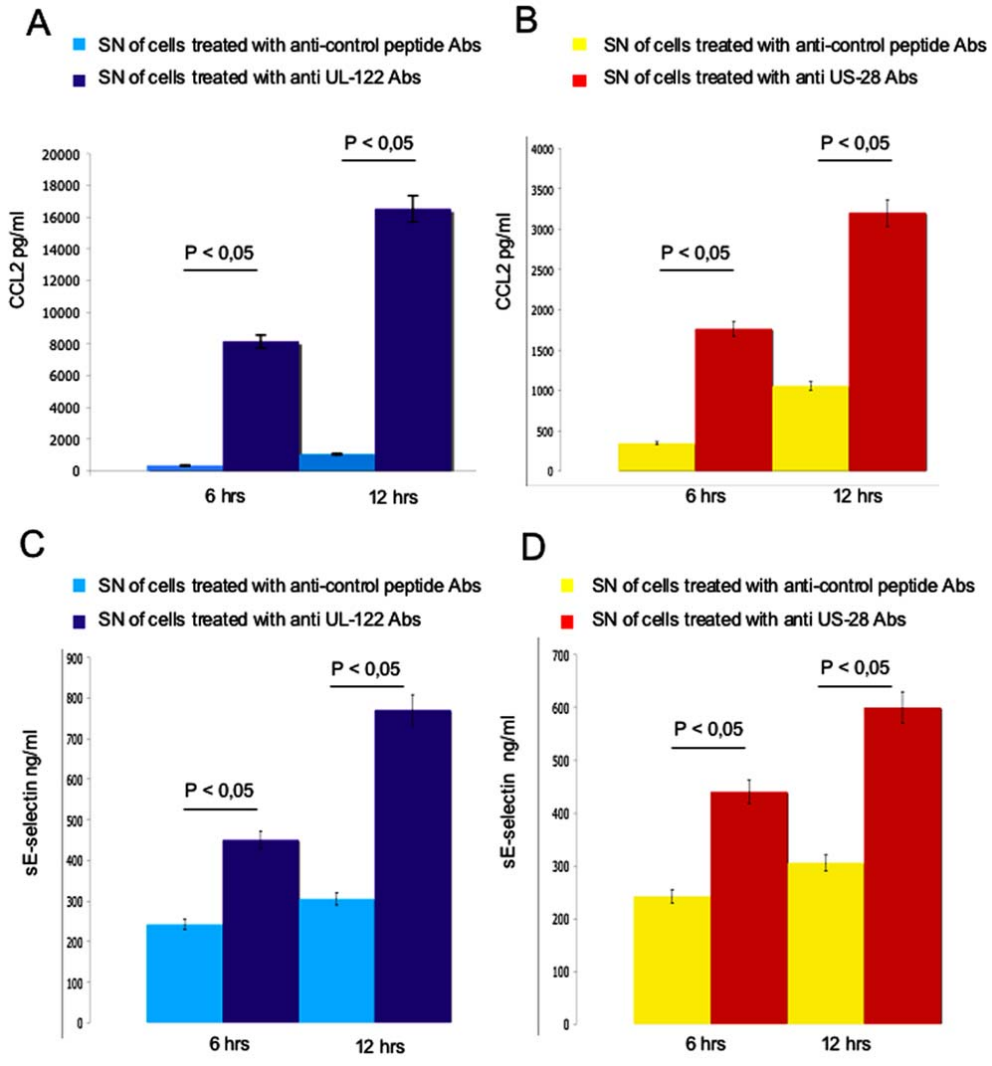
Soluble mediators released in cell culture supernatants. (A, B) Quantification of CCL2 released in the supernatant (SN) of HUVECs stimulated with antibodies (Abs) against the irrelevant peptide and with anti-UL122 and anti-US28 affinity purified antibodies. (C, D) Quantification of soluble E-selectin in the SN of HUVECs stimulated with abs against the irrelevant peptide and with anti–UL122 and anti-US28 affinity purified antibodies. Results are expressed in pg/mL and ng/mL, respectively. Results are expressed as mean of three independent experiments.

The gene array results were validated by real-time PCR of some of the genes up-regulated by both anti-US28 and UL122 antibodies (figure 6 A, B). These findings were paralleled by increased secretion of the corresponding molecules in the supernatant of stimulated cells (figure 7 A, B).

Using MedGene, the automated literature mining tool which summarizes and estimates the relative strength of all human gene disease relationships in Medline, the transcripts induced after stimulation with anti US28 antibodies were sorted into 3 classes, according to their relationship with the disease Atherosclerosis: 95 genes were directly associated to atherosclerosis, 348 were associated through others genes and 263 genes were new for this disease. Similarly, among the genes up-regulated by anti-UL122 antibodies, 40 genes resulted directly linked to atherosclerosis, 88 genes have been indirectly associated with the disease, and only 33 genes non associated to the atherosclerosis. Considering the down-regulated genes, 329 were not related to the disease after US28 antibody stimulation and 33 resulted not previously associated to the disease after UL122 antibody stimulation. A total of 25 genes were up-regulated by both anti-hCMV antibodies, while 26 genes were simultaneously down regulated by both anti-hCMV antibodies (Table S5 and Table S6). Interestingly all of these genes are classified as “directly related to atherosclerosis” when analyzed with MedGene.

We next wanted to identify the biological processes influenced by the engagement of CD49f and HSP60, connexin 45 and CD151 in HUVECs treated with either anti-US28 or anti-UL122 antibodies by using a pathway analysis software (Pathway Studio). To answer this question we generated connection pathways linking either CD151 and Connexin 45 or CD49f and HSP60 with the sets of modulated genes. The graphic outputs obtained showed that the different receptors use different pathways which ultimately lead to the same biological processes, including immune response, inflammation and apoptosis (Figure S1).

## Discussion

We had previously reported that antibodies isolated from patients with atherosclerosis and directed against the hCMV-derived proteins UL122 and US28 are able to induce endothelial cell apoptosis following the engagement of cell surface molecules [6]. The apoptotic process was more evident when HUVEC cells were exposed to anti-UL122 antibodies and here we show that these antibodies induce apoptosis earlier than anti-US28 antibodies do. Anti-US28 antibodies induce membrane translocation and release in the supernatant of HSP60. Abnormal surface expression of HSP60 sensitizes endothelial cells to apoptosis and cross-recognition of HSP60 on stressed endothelial cells by such antibodies may amplify the process. HSP60 is released in the supernatant and is able to trigger signaling through TLR4, suggesting a possible a link between the atherosclerotic process and defence against both foreign pathogens and endogenously generated inflammatory ligands [11]. Indeed TLR4 may play a pivotal role in the early stages of atherosclerosis since it is upregulated on endothelial cell by low shear stress and oxidized lipoproteins. On the other hand TLR4 initiates an immune response after recognition of pathogen-associated molecular patterns inducing upregulation of proinflammatory cytokines. HSP60, released upon stress signals, acts as an endogenous ligand for TLR4, thus amplifying TLR4 activity and suggesting a HSP60-related autocrine process [27]. Moreover it has been reported that ligation of TLR strongly inhibits cholesterol efflux from macrophages [28]. Therefore TLR4 may bridge oxidized lipoproteins, infection, inflammation and low shear stress in the pathogenesis of the disease [29].

To investigate the gene modulation induced by anti-hCMV antibodies, we used a gene array approach, which allows the simultaneous detection of thousands of genes in a given sample. By this approach we found that the purified anti-hCMV antibodies modulate a vast array of genes encoding for molecules which play a pivotal role in endothelial cells activation and damage. Interestingly the genes associated to atherosclerosis VCAM1, SELE, ICAM1, CCL2, CCL20, CXCL2, TAP1, NFKBIA, TCF4 and ABCG1 are upregulated in cells treated with both UL122 and US28. The up-regulation of genes encoding for the adhesion molecules ICAM-1, VCAM-1, E-selectin is present also when endothelial cells are stimulated by proinflammatory cytokines such as tumor necrosis factor alpha [30]. These adhesion molecules are markers of endothelial dysfunction and damage and have been found elevated in the sera of patients affected by atherosclerosis [31]. Reflecting the inflammatory status of the atherosclerotic process [32], different chemokine-encoding genes have been reported to be activated in endothelial cells [33]. Also in our experimental model, some chemokine-endoding genes were up-regulated, in particular the gene encoding for CCL2 presented the highest fold change, mainly in the cells treated with anti-UL122 antibodies. This result was confirmed by the high increase in CCL2 transcripts by Q-PCR and by the detection of increased levels of the molecule in the cell culture supernatants.

Genes involved in the extracellular matrix metabolism were also up-regulated reflecting the extracellular matrix rearrangement within the vessel walls during the atherosclerotic process [24]. The gene expression profile in our cells resulted in the up-regulation of genes encoding for several transcription factors and proteins involved in the intracellular signaling. In particular in cells stimulated with anti-US28 antibodies the genes encoding for ETS2 and calmodulin 1 were highly induced. In this context it has to be mentioned that different stress factors, (i.e. mechanical force and thrombin) may initiate intracellular signaling relevant to vascular damage [34]. Moreover, TANK and RELB induced in anti-UL122 treated cells, as well as MAPK induced by anti-US28 antibodies, are involved in the NF-kB activation, known to play a pivotal role in the regulation of several genes related to atherogenesis [25]. Finally the diminished expression of endothelial eNOS is an early event in endothelial cell damage and a suppressed eNOS activity has been recently reported in hCMV infected endothelial cells [35].

It is noteworthy that all of the genes modulated by the two subsets of anti-hCMV antibodies encode molecules that have been already associated with atherosclerosis [36], supporting the possible role of hCMV in inducing endothelial cell damage that plays a pivotal role in the atherosclerotic process.

Taken together these data indicate that the anti-UL122 and -US28 antibodies induce endothelial cell damage through the activation of genes encoding for adhesion molecules, chemokines, for molecules involved in immune response, inflammation and extracellular matrix deposition. Moreover anti-US28 antibodies ampliphy their effect on endothelial cells through the traslocation of HSP60 on cell surface.

Since these subset of anti-hCMV antibodies have been detected in the majority of patients with atherosclerosis [6], our results give further support to the role played by hCMV in the pathogenesis of the disease.

## Supporting Information

Figure S1 Functional pathway analysis of genes influenced by the engagement of CD151 and connexin 45 (A) and CD49f and HSP60 (B). The Pathway Studio software was used to identify connection pathways linking CD151 and connexin 45 or CD49f and HSP60 with the sets of modulates genes. Genes are represented as yellow ovals connected by arrows, major biologic processes related to these genes are represented as orange rectangles.
(0.29 MB TIF)

Table S1 Genes up-regulated by anti-US28 antibodies.
(0.17 MB XLS)

Table S2 Genes down-regulated by anti-US28 antibodies.
(0.26 MB XLS)

Table S3 Genes up-regulated by anti-UL122 antibodies.
(0.04 MB XLS)

Table S4 Genes down-regulated by anti-UL122 antibodies.
(0.03 MB XLS)

Table S5 Genes up-regulated by both anti-UL28 and anti-US122 IgG antibodies.
(0.02 MB XLS)

Table S6 Genes down-regulated by both anti-US28 and anti-UL122 antibodies.
(0.01 MB XLS)
