# Supplementary figures and images for: Endothelial Cells' Activation and Apoptosis Induced by a Subset of Antibodies against Human Cytomegalovirus: Relevance to the Pathogenesis of Atherosclerosis
Source: PLoS One. 2007 May 30;2(5):e473. doi: 10.1371/journal.pone.0000473 (PMC1868596; doi:10.1371/journal.pone.0000473)

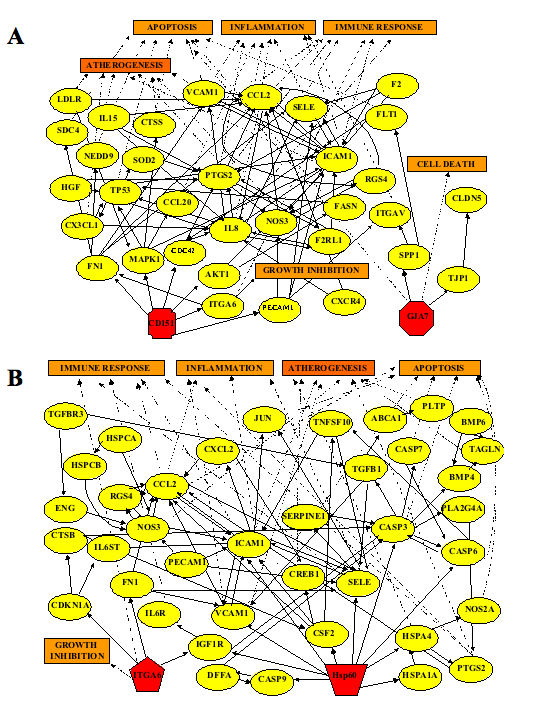

Supplement: Figure S1 — Functional pathway analysis of genes influenced by the engagement of CD151 and connexin 45 (A) and CD49f and HSP60 (B). The Pathway Studio software was used to identify connection pathways linking CD151 and connexin 45 or CD49f and HSP60 with the sets of modulates genes. Genes are represented as yellow ovals connected by arrows, major biologic processes related to these genes are represented as orange rectangles. (0.29 MB TIF) [file pone.0000473.s001.tif]
